# Efficacy of Erector Spinae Plane Block (ESPB) in pediatric cardiac surgeries: a systematic review and meta-analysis

**DOI:** 10.1016/j.bjane.2024.844579

**Published:** 2024-11-29

**Authors:** Verônica Pustrelo Damião, Priscila Pechim Andrade, Leonardo Saraiva Guimarães de Oliveira, Angélica de Fátima Assunção Braga, Vanessa Henriques Carvalho

**Affiliations:** a Universidade Estadual de Campinas (UNICAMP), Campinas, SP, Brazil; b Pontifícia Universidade Católica de Campinas (PUC-Campinas), Campinas, SP, Brazil; c Faculdade de Ciências Médicas de Minas Gerais (FCMMG), Belo Horizonte, MG, Brazil

**Keywords:** Cardiac surgery, Pediatrics, Regional anesthesia, Pain management, Sternotomy

## Abstract

**Background:**

Erector Spinae Plane Block (ESPB) effectively reduces pain scores for sternotomy in adults. However, evidence is insufficient to assert that the same result occurs in children. The aim of this systematic review and meta-analysis was to evaluate the efficacy of ESPB in pediatric cardiac surgeries.

**Methods:**

Systematic Medline, Embase and Cochrane searches were conducted for studies that compared ESPB versus no block or sham block for pediatric cardiac surgery under sternotomy. The primary outcome was cumulative opioid consumption for up to 48 hours. Statistical analyses were carried out with the use of RStudio version 1.2.1335. Heterogeneity was assessed by Cochran's Q test and I^2^ statistics. Quality assessment and risk of bias assessment complied with Cochrane recommendations.

**Results:**

Five studies, involving 328 patients (3 Randomized Controlled Trials [RCT], and 2 cohorts) were included. Of the 328 patients, 160 (48.7%) underwent ESPB. There were significant reductions in cumulative opioid consumption up to 48 hours after ESPB (SMD -0.68; 95% CI -1.13 – -0.23; p < 0.01). In the following outcomes ESPB failed to show superiority: postoperative nausea and vomiting (OR = 0.56; 95% CI 0.25–1.23; p = 0.54), fever (OR = 0.75; 95% CI 0.24–2.31; p = 0.58), length of intensive care unit stay in hours (MD -2.42; 95% CI -5.47–0.64; p < 0.01] and length of hospital stay in days (MD -0.87; 95% CI -2.69–0.96; p = 0.02). Only one cohort study had a high risk of bias.

**Conclusion:**

ESPB potentially reduces postoperative pain by significant reductions in cumulative opioid consumption up to 48 hours in pediatric cardiac surgery patients.

## Introduction

Enhanced Recovery After Surgery (ERAS) for cardiac surgery represents a comprehensive approach to cardiac surgical care, setting in motion multidisciplinary expertise and collaboration.^1^ Enhanced recovery protocols, originally developed for colorectal surgery, adapted to the context of cardiac surgery, have become increasingly prevalent across various clinical settings. In pediatric cardiac surgery, there is a strong emphasis on refining perioperative care methodologies to optimize postoperative recovery and alleviate the surgical stress response. Therefore, the use of multimodal opioid-sparing analgesia techniques, notably incorporating regional anesthesia approaches, has emerged as a prominent strategy.^2^

It has been highlighted that the Erector Spinae Plane (ESP) block is a promising regional anesthesia technique, used to provide effective analgesia in various surgical procedures. ESP block has been administered unilaterally in thoracic surgeries and bilaterally in cardiac surgeries. The advantage of the ESP block in comparison to other techniques, such as thoracic paravertebral and thoracic epidural blocks, is that it is easier and safer.^3^ Although regional anesthesia can be safely performed in children with a relatively low risk of complications,^4^ its utility remains relatively unexplored in pediatric cardiac surgery, particularly through a sternotomy.

Consequently, this meta-analysis seeks to compare the efficacy and safety of the Erector Spinae Plane (ESP) block versus no block or saline block in pediatric patients undergoing a sternotomy for cardiac surgery. By conducting a synthesis of available evidence, we aim to evaluate the impact of ESPB on opioid consumption, postoperative nausea and vomiting, fever, and length of Intensive Care Unit (ICU) and hospital stay.

## Methods

This systematic review followed the Preferred Reporting Items for Systematic Reviews and Meta-Analyses (PRISMA) guidelines.^5^

### Registration and protocol

The protocol was registered in the PROSPERO website (International Prospective Systematic Review Registry – Center for Comments and Dissemination at the University of York) on November 24,2023, under protocol CRD42023486534. https://www.crd.york.ac.uk/prospero/display_record.php?ID=CRD42023486534.

### Eligibility criteria

Eligibility criteria considered the classification of the acronym PICOS to answer the following focused question: ‘Is Erector Spinae Plane Block (ESPB) effective in Pediatric Cardiac Surgeries?’ P = Participants (Cardiac surgery with sternotomy in pediatric patients); I = Intervention (ESPB); C = Comparison (no block or saline block); O = Outcome (opioid consumption, nausea and vomiting, fever, length of intensive care stay and length of hospital stay); S = Study design (randomized and cohort studies).

### Inclusion criteria

Inclusion in this meta-analysis was restricted to studies that met all the following eligibility criteria: (1) Randomized or nonrandomized trials; (2) Comparison of the erector spinae plane block to no block or saline block and (3) Enrollment of patients under 18 years of age who underwent cardiac surgery through a sternotomy. In addition, studies were included only if they reported any clinical outcome of interest. Randomized, nonrandomized, quasi-experimental or pseudorandomized clinical trials, cohort studies, and cross-sectional studies were considered eligible. No studies based on population gender, language, or time of publication were excluded.

### Exclusion criteria

Exclusion criteria were, as follows: (1) No control group; (2) Patients over 18 years of age; (3) Experimental animal studies, reviews, expert opinions, letters to the editor, or any type of descriptive study.

### Information sources and search strategy

The search strategy was developed through a combination of words and appropriate truncations for each electronic database: EMBASE, PubMed/Medline and Cochrane Library. The terms were: ‘Children’, ‘child’, ‘infant’, ‘infants’, ‘pediatric’, ‘pediatrics’, ‘paediatric’, ‘paediatrics’, ‘ESP’, ‘ESPB’, ‘erector spinae’, ‘erector spinal’, ‘cardiac’, congenital’ and ‘sternotomy’. The search was performed on November 6, 2023, and updated on February 6, 2024. In addition, a manual search for references in articles included in the study was conducted. An expert who had not participated in the article selection process was also consulted to indicate potentially eligible articles.

### Selection process

Study selection occurred in two phases. In phase 1, two reviewers independently assessed the titles and abstracts of all references, excluding studies that failed to meet pre-established inclusion criteria. In Phase 2, the same reviewers independently read the full texts of the studies that had passed Phase 1, once again applying the same eligibility criteria. If the two reviewers disagreed, a third reviewer was brought in to resolve the conflict. In both phases the Rayyan website (http://rayyan.qcri.org) was used, which allowed references to be read independently and blindly between both reviewers.^6^

### Data collection process

Two reviewers collected data which was then discussed collectively. Data consisted of study characteristics, population characteristics, techniques performed, evaluation characteristics, result characteristics, and main conclusions. In case of any missing or incomplete data, the corresponding author was contacted once by e-mail to obtain the necessary information.^5^^,^^6^

### Data items

For a numerical outcome variable, the mean and standard deviation were collected. For categorical outcomes, the number of events of interest was recorded. All measurements in both the intervention and comparator groups were taken, including the sample size of each group.^5^^,^^6^

### Study risk of bias assessment

Observational studies were evaluated for methodological quality using the Joanna Briggs Institute critical appraisal tool.^7^^,^^8^ Two reviewers independently assessed each study, marking each criterion with a ‘yes’, ‘no’, ‘uncertain’, or ‘not applicable’. A high risk of bias was considered when the article reached a 49% ‘yes’, moderate when it ranged from 50% to 69%, and low when it exceeded 70%. Disagreements were solved by consulting a third reviewer. The risk of bias in randomized clinical trials was assessed by the Cochrane Collaboration Risk of Bias Tool, which evaluates seven domains: random sequence generation, allocation concealment, participant and professional blinding, result evaluator blinding, incomplete results, reports of selective results, and other sources of bias.^6^^,^^9^ To generate the figures, the Robvis web app (https://mcguinlu.shinyapps.io/robvis) was used.

### Effect measures

Continuous numerical variables assessed opioid consumption, calculating the Standard Mean Difference (SMD) between preoperative and postoperative scores. The Mean Difference (MD) measured the length of an Intensive Care Unit (ICU) stay and hospital stay. The Odds Ratio evaluated dichotomous outcomes, such as nausea, vomiting and fever.

### Synthesis methods

A meta-analysis with a random-effects model was performed using Studio version 1.2.1335 (RStudio Inc, Boston, USA). Study weights were determined by the Mantel-Haenszel method for binary variables and the inverse variance method for continuous variables. Variance (Tau^2^) was estimated using the restricted maximum likelihood method, and heterogeneity was assessed by the inconsistency Index (I^2^) and Cochran's Q test. For quantitative synthesis, it was required that at least three studies fulfilled the eligibility criteria and contained necessary data. The 95% Confidence Interval (95% CI) was calculated with a significance level set at 5%.^6^

### Reporting bias assessment

To reduce biases, we contacted the authors via email in search of missing data. Nevertheless, there was no response to the emails.

### Certainty assessment

The Grading of Recommendations, Assessment, Development, and Evaluation (GRADE) tool was employed to determine the certainty level of evidence for each specified outcome. Certainty assessment took into account several domains: risk of bias, inconsistency, imprecision, indirect evidence, and publication bias. Based on these factors, certainty of evidence was categorized into four potential levels: low, very low, moderate, or high.^10^

## Results

### Study selection and characteristics

**Figure 1:**
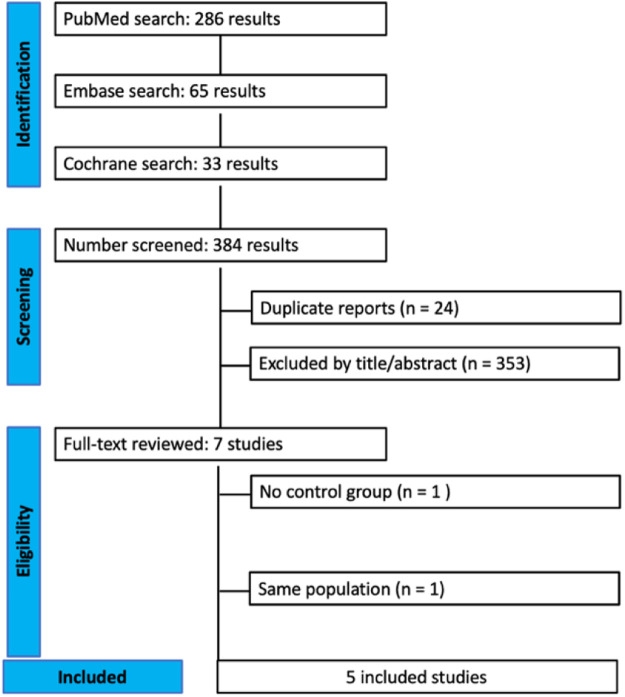
PRISMA flow diagram of study screening and selection. PRISMA, Preferred Reporting Items for Systematic Reviews and Meta-Analyses.

As detailed in Figure 1, the initial search yielded 384 results. After duplicate records and ineligible studies were removed, 7 studies remained and were fully reviewed based on inclusion criteria. Two studies were excluded: duplicate data in a study conducted by Karacaer et al.^11^ and lack of a control group in another study carried out by Macaire et al.^12^ A total of 5 studies were included, comprising 328 patients from 3 randomized controlled trials (RCTs),^11^^,^^13^^,^^14^ one Prospective Cohort Study (PCS),^15^ and one Retrospective Cohort Study (RCS).^16^ All studies were published in the last five years. A total of 160 (48%) patients received ESPB and 168 (52%) did not receive any block. Study characteristics are reported in Table 1. Significant variability existed between studies: age of participants (range: 3 to 153 months), weight of participants (range: 3.6 to 57.6 kilograms), and duration of follow-up periods; local anesthetic used; concentration, dose, and volume of anesthetics, as well as puncture site.

**Table 1:** Characteristics of studies included in the meta-analysis.

Estudo	Study type	Country	Number of patients		Age (mo): Mean ± SD or Median (IQR)		Weight (kg): Mean ± SD or Median (IQR)		Male Sex, n (%)		Follow-up (hours)	Local Anesthetic	Volume of local anesthetic by side	Total dose of local anesthetic	Transverse process level
			ESP	NB	ESP	NB	ESP	NB	ESP	NB					
Kaushal, 2019	RCT	Índia	40	40	28.43±21.5	29.83±24.07	10.81±5.51	9.47±4.86	22 (55%)	23 (57.5%)	12	Ropivacaine 0.2%	0.75 mL.kg^-1^	3 mg.kg^-1^	T3
Roy, 2020	PCS	USA	10	20	108 (69.6‒135.6)^b^	111.6 (58.8-153.6)^b^	31.5 (20.8‒49.9)	31.2 (17.4‒57.6)	4 (40%)	8 (40%)	96	Ropivacaine 0.2% + Licocaine 1%	0.375 mL.kg^-1^ Ropivacaine 0.2% + 0.05 mL.Kg^-1^ Licocaine 1% + catheter aend of surgey	1.5 mg.kg^-1^ Rovivacaine 0.2% + 1 mg.kg^-1^ Licocaine 1% + catheter aend of surgey	T4/T5
Gado, 2022	RCT	Egypt	50	48	28.2±25.3	33.5±24.9	11.7±7.3	13±7	22 (44%)	21 (43.8%)	24	Bupivacaine 0.25%	0.4 mL.kg^-1^	2 mg.kg^-1^	T5
Karacaer, 2022	RCT	Turkiye	20	20	72±27.36^b^	72±31.68^b^	19.9±8.33	20.68±8.84	11 (55%)	10 (50%)	24	Bupivacaine 0.25%	0.5 mL.kg^-1^	2.5 mg.kg^-1^	T4/T5
Cruz-Suarez, 2023	RCS	Colombia	40	40	43.4±57.56	45.59±67.03	9.5 (6.1‒15.5)	6 (3.65‒21.55)	18 (45%)	17 (42.5%)	48	Bupivacaine 0.125%	0.7 mL.kg^-1^^a^end of surgey	1.75 mg.kg^-1^^a^ end of surgey	T4/T5

ESP, Erector spinae block; NB, No block or Saline block; RCT, Randomized clinical trial; PCS, Prospective cohort study; RCS, Retrospective cohort Study.

a Median converted values

b Original data in Years.

### Pooled analysis of all studies

**Figure 2:**
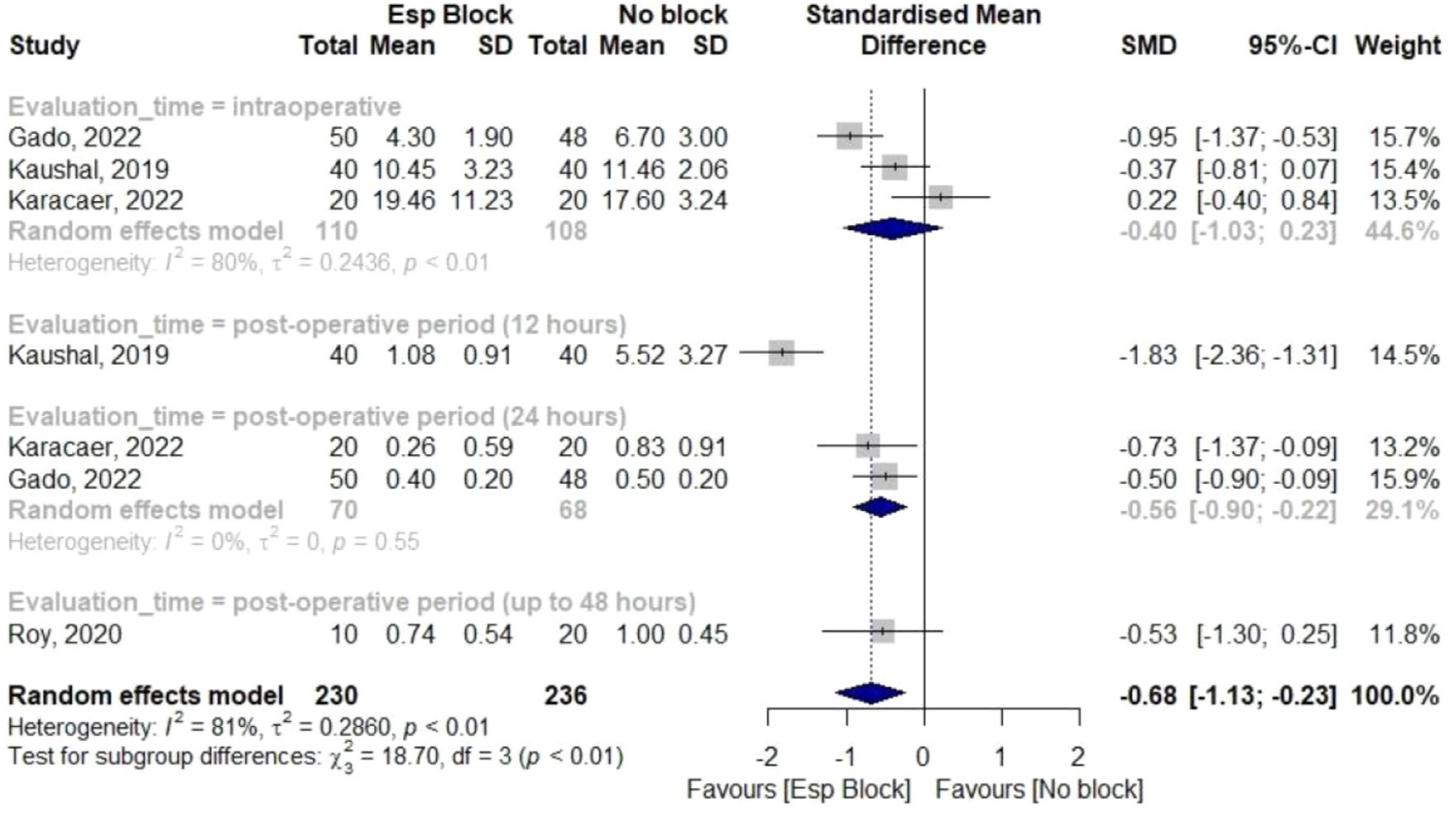
Forest plot for opioid consumption.

In those receiving ESPB, there was an overall trend towards decreased pain scores and significantly lower opioid consumption. Four studies showed a significant reduction in cumulative opioid consumption up to 48 hours [SMD -0.68; 95% CI -1.13 – -0.23; p < 0.01; I^2^ = 78%; Fig. 2].

Pain scores were measured in all studies, but the measurements taken were not suitable for comparison. In addition to different pain scales, two studies assessed pain 6 hours after surgery, three studies assessed pain after 12 hours, but only two of these studies had sufficient data for analysis. Two studies assessed pain 24 hours after surgery, though only one had sufficient data. As a result, although a decrease in pain scores was suggested in the studies, plotting this outcome was not possible.

**Figure 3:**
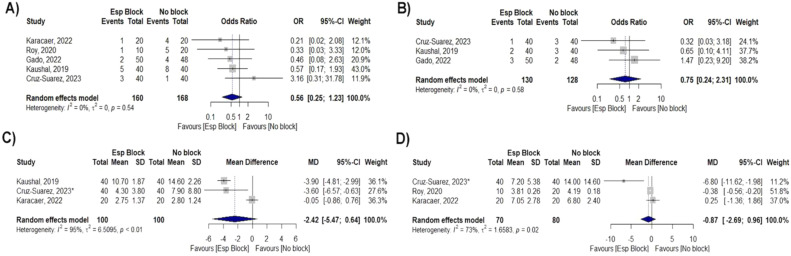
Forest Plot for secondary outcomes. (A) Nausea and vomiting, (B) Fever, (C) Length of ICU stay and (D) Length of hospital stay.

All included studies evaluated the occurrence of nausea and vomiting in the postoperative period, and there was no significant reduction between groups (OR = 0.56; 95% CI 0.25–1.23; p = 0.54; I^2^ = 0%; Fig. 3A).

Three studies showed no difference in the occurrence of fever (OR = 0.75; 95% CI 0.24–2.31; p = 0.58; I^2^ = 0%; Fig. 3B). Three studies showed no difference in length of ICU stay (MD -2.42; 95% CI -5.47–0.64; p < 0.01; I^2^ = 95%; Fig. 3C), and hospital length of stay (MD -0.87; 95% CI -2.69–0.96; p = 0.02; I^2^ = 73%; Fig. 3D).

### Sensitivity analysis

The leave-one-out analysis did not bring any notable differences compared to the main analysis regarding postoperative opioid consumption, as shown in Supplementary Figure 5.

### Reporting biases

**Figure 4:**
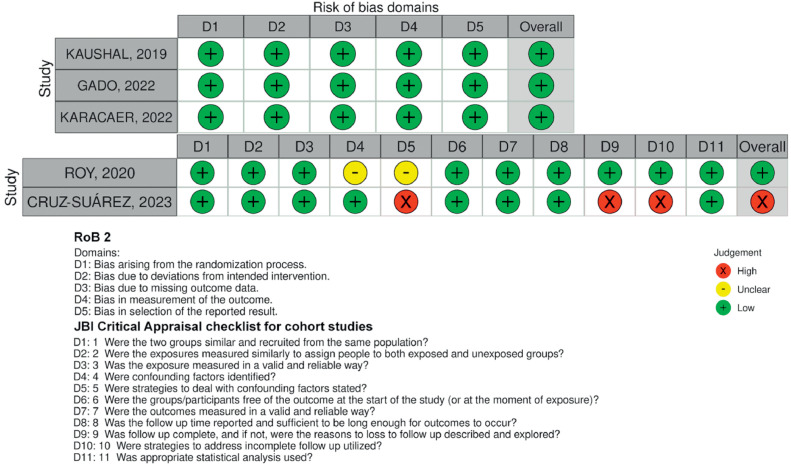
Risk of bias assessment by ROB2 and JBI Critical appraisal tool.

Individual RCT and non-RCT appraisals are reported in Figure 4. Three randomized studies matched intervention and control patients according to baseline characteristics.^11^^,^^13^^,^^14^ One of the non-RCT studies had no evidence suggestive of publication bias,^15^ while the other^16^ had a high risk of publication bias due to the lack of strategies to deal with confounding factors and incomplete follow-up without any explanation.

### Quality assessment

The certainty of evidence assessed by the GRADE tool was considered low for opioid consumption and very low for other outcomes. As shown in Table 2, the domains responsible for the decrease in the certainty of evidence correlated with inconsistency (I^2^ > 50% in outcomes of opioid consumption, length of ICU stay and hospital length of stay), caused by a high level of heterogeneity in the study population and correlated with imprecision, due to the wide CI in all outcomes.

**Table 2:** Summary of findings table (GRADE).

Certainty assessment							No of patients		Effect		Certainty	Importance
No of studies	Study design	Risk of bias	Inconsistency	Indirectness	Imprecision	Other considerations	ESPB	No Block	Relative (95% CI)	Absolute (95% CI)		
**Opioid consumption (assessed with: standard mean difference)**												
3	Randomised trials	Not serious	Serious^a^	Not serious	Serious^b^	None	466	466	‒	SMD **0.68 SD lower** (1.13 lower to 0.23 lower)	⨁⨁○○ Low	Critical
**Nausea and vomiting (assessed with: Odds Ratio)**												
5	Non-randomised studies	Not serious	Not serious	Not serious	Serious^c^	None	12/160 (7.5%)	22/168 (13.1%)	**OR 0.56** (0.25 to 1.23)	**53 fewer per 1.000** (from 95 fewer to 25 more)	⨁○○○ Very low	Important
**Fever (assessed with: Odds Ratio)**												
3	Non-randomised studies	Not serious	Not serious	Not serious	Serious^c^	None	6/130 (4.6%)	8/128 (6.3%)	**OR 0.75** (0.24 to 2.31)	**15 fewer per 1.000** (from 47 fewer to 71 more)	⨁○○○ Very low	Important
**Length of ICU stay (assessed with: mean difference)**												
3	Non-randomised studies	Not serious	Serious^a^	Not serious	Serious^d^	None	100	100	‒	MD **2.42 lower** (5.47 lower to 0.64 higher)	⨁○○○ Very low	Important
**Length of hospital stay**												
3	Non-randomised studies	Not serious	Serious^a^	Not serious	Serious^d^	None	70	80	‒	MD **0.87 lower** (2.69 lower to 0.96 higher)	⨁○○○ Very low	Important

CI, Confidence Interval; MD, Mean Difference; OR, Odds Ratio; SMD, Standardized Mean Difference.

Explanations:

a The severity of cardiac disease in the population was not clear in all studies, as well as the classification by the American Society of Anesthesiologists (ASA), which may cause variability in the effect estimate.

b The samples from the studies in question were relatively small, leading to significant variability in precision.

c All studies presented a wide confidence interval, possibly due to the outcome having low frequency.

d The sample size and the potential heterogeneity of the population made the outcome imprecise.

## Discussion

Our meta-analysis showed a reduction in total opioid consumption up to 48h, in alignment with the ERAS protocol, associated with lower postoperative opioid use. This result was confirmed by decreased pain scores, in agreement with several authors.^11^^,^^13^, ^14^, ^15^, ^16^ With a rise in ERAS protocol publications, there has been growing interest in the study and investigation of fascial plane blocks to optimize perioperative resources in terms of quality and cost-effectiveness. Pain scales, opioid consumption and side effects are consistent with patient's perception of care. Meanwhile, time to extubation, and length of ICU stay or regular ward, directly reflect operational costs. Within this perspective, ESPB could play an important role in ERAS protocol improvement in pediatric cardiac surgery.^2^^,^^17^

Intraoperative opioid consumption was reduced up to 48 hours after surgery (SMD -0.68), suggesting that ESPB may mitigate neuroendocrine metabolic and immunological response to surgical trauma. These responses are recognized as significant risk factors for the development of mental disorders.^18^

Although opioid consumption was shown to be decreased, there was no significant reduction between groups when nausea and vomiting, length of ICU stay, and hospital stays were analyzed. Furthermore, the analysis of fever did not differ between groups. Despite the low heterogeneity of outcomes (nausea, vomiting, and fever), the few events and small sample size could explain the lack of difference in nausea, vomiting, and fever in both groups.

Overall, this meta-analysis encompassed four studies that had a low risk of bias and just one retrospective cohort with a high risk of bias. It shows that ESPB has a crucial role in outcome improvement in pediatric cardiac patients due to its ease of performance, absence of a central neuraxial route, possibility of catheter insertion away from the surgical field, reducing the potential risk of spinae or epidural hematomas, and wound infection that is associated with other regional anesthesia techniques, as evidenced by adult sternotomies for cardiac surgery. However, due to the high level of heterogeneity and imprecision in most of the outcomes, the certainty of evidence was considered low for opioid consumption and very low for our secondary outcomes, highlighting the need for quality assessment which was not explored in a recent meta-analysis for the adult population.^17^

This study has some limitations. In this meta-analysis, a high level of heterogeneity was observed due to the performance of ESPB (technical aspects, different thoracic puncture sites, difference in total volume and concentration of local anesthetics between studies) and variability characteristics of the pediatric population between studies, mainly age and weight. Moreover, the low number of studies conducted and low sample size highlight the need for further evidence-based studies to find and share generalizable results. Due to a lack of homogeneity of pain score scale measurements between studies, it was not possible to gather outcome data, although reduced opioid consumption, as a surrogate marker, can potentially resemble lower pain scores.

Inconsistency in block performance in the studies and the small number of patients precluded an optimal comparison. Nevertheless, it is possible to envision a future where superficial blocks can gain prominence in pediatric cardiac surgery. It may also be a cost-effective way to improve the postoperative experience.

## Conclusion

ESPB in pediatric cardiac surgery was associated with a significant reduction in postoperative opioid requirements. There was no difference in nausea and vomiting, fever, length of ICU stay or length of hospital stay. The lack of large randomized controlled trials and the high heterogeneity among studies suggest the need for further studies to ensure the effectiveness of ESPB in pediatric cardiac surgery patients.

## Availability of data, code and other materials

This study was conducted using data extracted from previously published trials, and the authors do not have access to patient-level data from individual studies. We recommend contacting the corresponding author of each study for further requests. The datasets used for this meta-analysis are available from the corresponding author upon reasonable request.

## Registry of the study

PROSPERO website (International Prospective Systematic Review Registry – Center for Comments and Dissemination at the University of York) under protocol CRD42023486534, on November 24, 2023. https://www.crd.york.ac.uk/prospero/display_record.php?ID=CRD42023486534.

## Authors’ contributions

Verônica Pustrelo Damião: Conception and design of the study; acquisition of data, analysis and interpretation of data; drafting the article and revising it critically for important intellectual content.

Priscila Pechim Andrade: Acquisition of data, analysis and interpretation of data; drafting the article and revising it critically for important intellectual content.

Leonardo Saraiva Guimarães de Oliveira: Acquisition of data, analysis and interpretation of data; drafting the article and revising it critically for important intellectual content.

Angélica de Fátima Assunção Braga: Analysis and interpretation of data; drafting the article and revising it critically for important intellectual content.

Vanessa Henriques Carvalho: Conception and design of the study, acquisition of data, analysis and interpretation of data; drafting the article and revising it critically for important intellectual content.

## Conflicts of interest

The authors deny any conflicts of interest. This research did not receive any specific grant from funding agencies in the public, commercial, or not-for-profit sectors.

## Appendix Supplementary materials

Image, application 1

## Footnotes

Supplementary material associated with this article can be found in the online version at doi:10.1016/j.bjane.2024.844579.
