# Supplementary material for: Efficacy of Erector Spinae Plane Block (ESPB) in pediatric cardiac surgeries: a systematic review and meta-analysis
Source: Braz J Anesthesiol. 2024 Nov 29;75(2):844579. doi: 10.1016/j.bjane.2024.844579 (PMC11719831; doi:10.1016/j.bjane.2024.844579)

**BJAN-D-24-00334_Supplementary material**

**Supplementary material**

**Figure 5** Sensitivity analysis: postoperative opioid consumption.


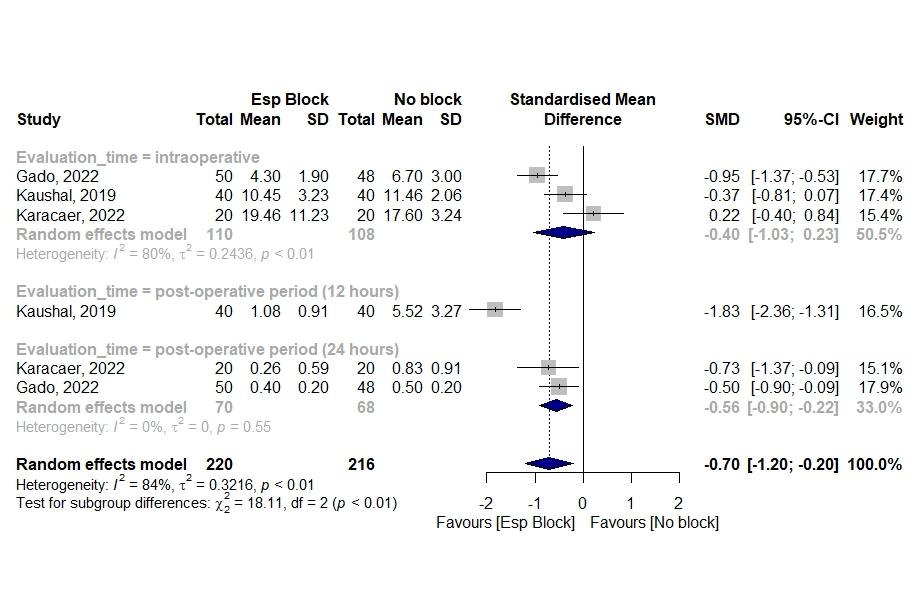

Supplement: Supplementary file 1 [file mmc1.docx]
